# Supplementary material for: Chromothripsis during telomere crisis is independent of NHEJ, and consistent with a replicative origin
Source: Genome Res. 2019 May;29(5):737–49. doi: 10.1101/gr.240705.118 (PMC6499312; doi:10.1101/gr.240705.118)
Supplement: Supplemental Material [file supp_gr.240705.118_Supplemental_file_1.zip › contigs/annotated_contigs/DB91/contig.3.DB91_length_285_mean_cov_6.0350877193.docx]

**DB91_length_285_mean_cov_6.0350877193**

```
TCCCACCTCAGCCTCCTGAGTAGCTGGTACTACAGGTGCACACCAACACACTCAGCTAATTTT|CT|TTTATTTTTTCTTTTCTTTTCT
 >chr15:58199492-58199555 - E=2e-17                               >chr7:90096543-900965
TTTCTTTTCTTT|T|TTTCTTTTCTTTTCTTTCTTCTTTCTTTCTTTCTTCTTTTTCTTTTCTTTCTCTTTCTTTTTTTCTTCTCTCTC
78 - E=1e-09 p=0e+00 >chr4:61223873-61224007 + E=2e-66
TCTTTCTTTCCTTCCTTCCTTCCTTCTTTCTCTCTCTCTTTTTCCTTTTCTTTCTTTCT|GTCTTAGATATACATAGACACCGGACTTA

ATGAAGTCTACAATCATATAAGG
```
